# Real-Life Performance and Clinical Outcomes of Portico Transcatheter Aortic Valve with FlexNav Delivery System: One-Year Data from a Single-Center Experience

**DOI:** 10.3390/jcm12165373

**Published:** 2023-08-18

**Authors:** Abdullah Yildirim, Omer Genc, Emre Pacaci, Omer Sen, Ibrahim Halil Kurt

**Affiliations:** 1 Department of Cardiology, Adana City Training & Research Hospital, University of Health Sciences, 01230 Adana, Turkey; emre.pcci@hotmail.com (E.P.); kardiyosen@gmail.com (O.S.); ibrahimhalilkurt@gmail.com (I.H.K.); 2 Department of Cardiology, Basaksehir Cam & Sakura City Hospital, 34480 Istanbul, Turkey; dr.genc@hotmail.com

**Keywords:** TAVR, portico, FlexNav, VARC-3, mortality, paravalvular leak, self-expandable

## Abstract

Significant progress has been made in both valves and delivery systems (DSs) for transcatheter aortic valve replacement (TAVR) procedures. We aimed to present one-year real-life data regarding TAVR procedures using Portico transcatheter heart valves (THVs) with new-generation, low-profile FlexNav DSs. This retrospective, single-center study enrolled 169 consecutive patients (mean age: 75.8 years, 68% females) with severe aortic stenosis undergoing TAVR with Portico THVs and FlexNav DSs between 2020 and 2021. We evaluated safety and efficacy outcomes, following the VARC-3 consensus, periprocedurally and at 30 days and 1 year. Procedural success was observed in 95.9% of cases, and no procedural mortality occurred. At 30 days, the rates of all-cause mortality, cardiovascular mortality, and neurological events were 4.7%, 3.6%, and 3.0%, respectively. Additionally, major vascular complications, acute kidney injury, and bleeding were recorded at rates of 11.2%, 14.8%, and 7.7%, respectively. The 1-year data showed all-cause mortality, cardiovascular mortality, and neurological event rates of 10.7%, 8.3%, and 7.7%, respectively. The moderate paravalvular leak and permanent pacemaker rates at 1 year were 2.6% and 12.2%, respectively. This real-life data provided evidence of positive outcomes and high technical success with Portico THVs and FlexNav DSs. Furthermore, we found low rates of mortality and neurological events, with satisfactory hemodynamic and functional results.

## 1. Introduction

Since Alain Cribier performed the first transcatheter aortic valve replacement (TAVR) procedure in 2002 [1] to treat severe aortic stenosis (AS), more than 800,000 implantations have been carried out in 65 countries. Initially reserved for high- and extreme-risk patients, TAVR has emerged as an alternative to surgical aortic valve replacement (sAVR), even for AS patients with low to moderate surgical risk [2,3,4,5,6], following non-inferior findings in clinical research [7]. The success of TAVR has been attributed not only to advancements in valve designs but also to improvements in delivery systems (DSs), which have addressed challenges such as the complex peripheral anatomy and horizontal aorta, particularly in making the transfemoral route, the primary access route in TAVR procedures, available to a larger patient population [8]. The introduction of the Portico™ (Abbott Structural Heart, Minneapolis, MN, USA) transcatheter heart valve (THV) with the FlexNav DS (Abbott Structural Heart, Minneapolis, MN, USA) in 2019 aimed to enhance the safety and ease of TAVR procedures with THVs, leading to optimized outcomes. The FlexNav DS, which received clinical approval from the Food and Drug Administration (FDA) in 2021, features a smaller diameter (14–15 French equivalent), integrated sleeve, hydrophilic coating, and controlled release [9].

To standardize and define endpoints for clinical research in valvular heart diseases, the Valve Academic Research Consortium (VARC) was established in 2010. The rapid evolution of the TAVR process and increased experience led to the release of the updated VARC-2 consensus report in 2012 [10,11]. As TAVR procedures expanded to include low-risk patients and device technology advanced secondary outcomes, such as neurological events, paravalvular leak (PVL), hemorrhage, and vascular complications, gained significance. In response to these advancements, the Valve Academic Research Consortium-3 (VARC-3) criteria [12] were introduced in 2021, enabling uniform clinical definitions. This study aimed to assess safety and efficacy metrics at the procedural, 30 day, and 1 year intervals, as well as the hemodynamic and functional consequences, in severe AS patients who underwent TAVR using the Portico THV and FlexNav DS following the VARC-3 criteria.

## 2. Materials and Methods

### 2.1. Study Population and Design

This retrospective, single-center study at Adana City Training and Research Hospital included 175 consecutive symptomatic patients with severe AS who underwent treatment with the Portico THV and FlexNav DS between June 2020 and December 2021 based on data extracted from the hospital registry. After excluding patients who underwent TAVR-in-TAVR or TAVR-in-sAVR procedures (*n* = 2) and those with significant missing data (*n* = 4), a total of 169 patients were included in the subsequent analysis. Patient data were collected by two cardiologists who were blinded to the study design. The study included subgroups of AS based on aortic valve area (AVA) ≤ 1.0 cm^2^ and indexed AVA ≤ 0.6 cm^2^/m^2^ and further categorized high-gradient AS (mean transvalvular gradient (MTVG) ≥ 40 mm Hg, transvalvular peak velocity ≥ 4.0 m/s) and low-gradient AS (MTVG < 40 mm Hg, transvalvular peak velocity < 4.0 m/s) [13]. The decision to perform TAVR was made by a heart team comprising a cardiologist, invasive cardiologist, cardiovascular surgeon, and anesthesiologist, taking into consideration the Society of Thoracic Surgeons Predicted Risk of Operative Mortality (STS PROM) score, vulnerabilities and comorbidities not evaluated by surgical risk calculators (such as porcelain aorta, history of radiotherapy, etc.), and the regulations of the Republic of Turkey Ministry of Health. The TAVR procedures were performed by experienced operators (I.H.K. and O.S.), both of whom had completed the Portico THV implantation training program and performed a minimum of 50 operations per year.

### 2.2. Evaluation and Procedures

All study participants underwent multislice computed tomography to determine valve size (23, 25, 27, and 29 mm). The study included patients with native annulus sizes ranging from 19 to 27 mm who were treated with the Portico THV. The right common femoral artery was the preferred access route for all procedures; however, the left common femoral artery was used when the right side was unsuitable. The anatomy of the iliofemoral arteries was assessed using multislice computed tomography and traditional angiographic imaging. In cases where the iliofemoral route was unsuitable to achieve the required ≥5.0 mm diameter for the 14 French FlexNav DS-SM in 23–25 mm valve implantation, the ≥5.5 mm diameter required for the 15 French FlexNav DS-LG in 27–29 mm valve implantation—or, if there were limitations to bilateral femoral access, the subclavian/axillary route—was preferred. A suture-mediated percutaneous closure device, the Perclose ProGlide (Abbott Structural Heart, Minneapolis, MN, USA), was used for closure, including in cases involving the subclavian/axillary route. Since the FlexNav DS includes an integrated sheath with a hydrophilic coating, routine sheath insertion was not performed. Rapid pacing was employed during balloon pre- and post-dilatation, but routine rapid pacing was not performed during valve replacement. Echocardiographic examinations were conducted by independent and experienced physicians at the imaging center of the clinic using the ACUSON SC2000 Prime (Siemens Medical Solutions, Mountain View, CA, USA) echocardiography device before implantation, as well as at the 1st month and 12th month following implantation. According to the most recent recommendations, the heart’s chambers and functions, valves, ejection fraction, and other echocardiographic data were assessed. Based on the continuity equation, AVA was calculated [14].

### 2.3. Endpoints

All primary and secondary endpoints in the present study were defined according to the VARC-3 criteria [12]. The primary endpoints included 1-year all-cause mortality, cardiovascular mortality, and neurological events. The secondary endpoints encompassed new permanent pacemaker (PPM) implantation, myocardial infarction (MI), bleeding, hemodynamic results, atrial fibrillation (AF), and overall vascular complications at the 1-year follow-up. The Neurologic Academic Research Consortium (NeuroARC) consensus, recommended by VARC-3, was utilized for classifying neurological events specific to cardiovascular interventions [15]. The study included symptomatic, permanent NeuroARC type-1 events (ischemic and hemorrhagic stroke, hypoxic-ischemic injury), type-2 events (covert central nervous system infarction or hemorrhage), and type-3a events (transient focal-type signs or symptoms), while excluding type-3b events (temporary, no evidence from neuroimaging, or without imaging performed), such as delirium. Acute kidney injury was defined in accordance with the Kidney Disease: Improving Global Outcomes (KDIGO) guideline [16]. This classification encompassed all stages from stage 1 (1.5–2.0-fold increase within seven days or ≥0.3 mg/dL within 48 h) to stage 4 (new temporary or permanent renal replacement therapy). The diagnosis of MI was made using the VARC-3-modified criteria [17,18,19]. The study included type-2 (major), type-3 (life-threatening), and type-4 (leading to death) bleeding events according to VARC-3, while excluding type-1 (minor) bleeding events.

### 2.4. Statistical Analysis

Data analysis was performed using the Statistical Package for the Social Sciences (SPSS) Statistics for Windows, Version 25.0 program (IBM Corp., Armonk, NY, USA). The normality distribution of continuous variables was assessed through both analytical methods (Kolmogorov–Smirnov test) and visual methods (histograms and probability plots). Continuous variables are reported as the mean ± standard deviation or median (interquartile range; 25th–75th percentiles) based on their distribution pattern. Categorical variables were compared using the χ2 test or Fisher’s exact test, and the results are presented as numbers (n) and percentages (%). Paired Student’s t-tests were used to compare the changes between the 30th day and 1st year for echocardiographic AVA and MTVG measurements, while the Wilcoxon signed-rank test was employed to assess the change in NYHA functional class. Kaplan–Meier plots, log-rank tests, and Cox proportional hazard models were utilized for time-to-event analysis of the results (mortality, STS PROM score, PPM rates, and neurological events). Proportional-hazards assumptions were tested based on the Schoenfeld residuals and visual inspections of the log-log plots. All statistical analyses were two-sided, and a significance level (alpha) of 0.05 was used for all tests.

## 3. Results

### 3.1. Baseline Characteristics

The study population had a mean age of 75.8 ± 7.7 years, with 68.0% of the participants being female. The mean STS PROM score was 4.63 ± 1.73. Most individuals (56.2%) were classified as NYHA functional class III, while 4.7% were in NYHA class IV. The most prevalent comorbidity was hypertension (53.8%), followed by diabetes mellitus (28.5%) and smoking (19.5%). Malignancy (3.0%) was the least frequently observed comorbidity. Peripheral arterial disease was present in 17.8% of participants prior to the procedure, and 3.6% had a pre-existing PPM. Baseline laboratory parameters showed an estimated glomerular filtration rate of 68 mL/min/1.73 m^2^ and a hemoglobin value of 11.5 mg/dL. Transthoracic echocardiographic measurements revealed AVA, left ventricular ejection fraction, and MTVG values of 0.62 ± 0.16 cm^2^, 53.9 ± 10.2%, and 50.2 ± 13.3 mm Hg, respectively. Bicuspid aortic valve morphology was observed in 17.2% of patients. Table 1 represents the detailed demographic, laboratory, and echocardiographic characteristics.

### 3.2. Procedural and 30-Day Outcomes

The procedure was performed under local anesthesia/conscious sedation (LA/CS) for 97% (*n* = 164) of the study population. Valve size preference rates were 3.5% for 23 mm, 14.2% for 25 mm, 22.5% for 27 mm, and 59.8% for 29 mm. Re-sheathing was required in 84.0% (*n* = 142) of cases, with 39 (27.5%) patients requiring three or more re-sheathing/repositioning procedures. A single Portico THV was successfully implanted in 162 (95.9%) patients. In 7 (4.1%) patients, a second Portico THV of the same size as the first valve was implanted due to pop-up into the ascending aorta. No migration to the left ventricular outflow tract was observed. Pre- and post-dilatations were performed in 79.8% and 52.6% of patients, respectively. The transfemoral approach was used in 98.2% of cases, while the remaining 3 (1.8%) patients underwent the left subclavian/axillary route. Two patients required a stiffer wire due to challenging aortic architecture. No procedural mortality, annular rupture, or conversion to open-heart surgery occurred. The concomitant coronary intervention rate was 6.5%. The procedural success rate was 95.9%, while the technical success rate was 88.8%. Detailed procedural data can be found in Table 2.

When examining the postprocedural 30-day endpoints in detail, the rates of all-cause mortality, cardiovascular mortality, and neurological events were found to be 4.7%, 3.6%, and 3.0%, respectively. Periprocedural myocardial infarction was diagnosed in 1.2% patients. The rate of new PPMs was 11.0%. While 87.0% of the patients did not experience any peripheral/access site-related issues, major vascular complications were detected in 11.2% of the cases. The occurrence of major/life-threatening bleeding was observed in 12.4% of the patients, but none of these cases resulted in death (Table 3).

### 3.3. One-Year Outcomes and Timing and Causes of Mortality

**Figure 1:**
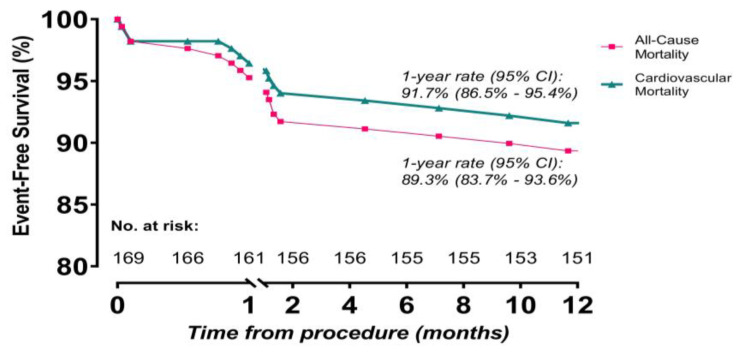
Kaplan–Meier survival rates for overall and cardiovascular deaths at 1–year follow-up.

**Figure 2:**
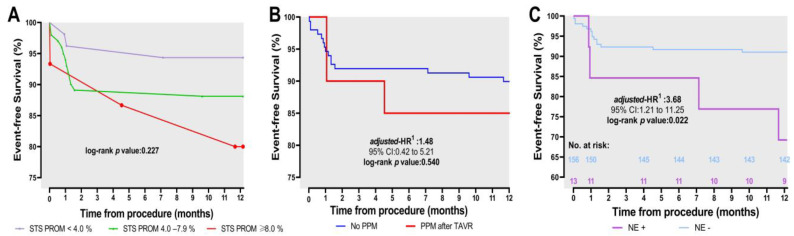
Kaplan–Meier survival rates for all-cause deaths by STS PROM score (**A**), implantation of PPM (**B**), and presence of neurological events (**C**), separately. ^1^ Note that the data have been adjusted for age and sex. Abbreviations—NE: neurological event, PPM: permanent pacemaker, STS PROM: Society of Thoracic Surgeons Predicted Risk of Operative Mortality. *p*-value of <0.05 was considered statistically significant.

At the 1-year follow-up, the primary endpoints—namely, all-cause mortality, cardiovascular mortality, and neurological events—were observed at rates of 10.7%, 8.3%, and 7.7%, respectively. When evaluating the secondary endpoints, the rate of myocardial infarction was found to be 1.3%. The incidence of serious bleeding was 15.4%. Additionally, 13.0% of the patients experienced vascular challenges, and 12.2% required new PPMs (Table 4). Bleeding emerged as the primary cause of cardiovascular mortality at both one month and one year, with rates of 1.2% and 1.8%, respectively, followed by heart failure, sudden cardiac death, and death of unknown causes (Table 5). Periprocedural mortality occurred in 5.3% of the patients, while early mortality was observed in 5.9% of the cases. The Kaplan–Meier event rates for all-cause mortality and cardiovascular mortality were calculated as 10.7% (95% confidence interval (CI): 6.4% to 16.3%) and 8.3% (95% CI: 4.6% to 13.5%), respectively (Figure 1). The occurrence of a PPM (log-rank *p* value: 0.540) (Figure 2A) and the categorized STS score (log-rank *p* value: 0.227) (Figure 2B) did not exhibit a significant association with death, whereas neurological events showed a significant impact on all-cause mortality (adjusted hazard ratio: 3.68, log-rank *p* value: 0.022, 95% CI: 1.21 to 11.25) (Figure 2C).

### 3.4. Hemodynamic and Functional Assessments

**Figure 3:**
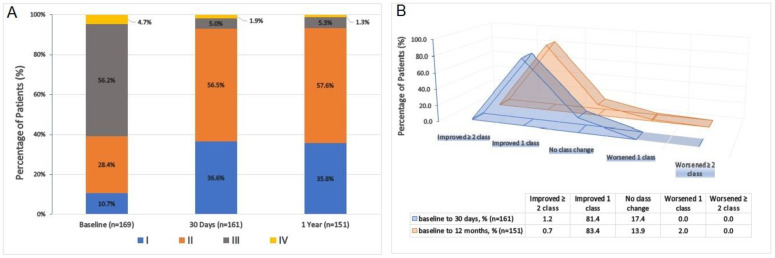
Changes in New York Heart Association (NYHA) class after TAVR: (**A**) patients’ NYHA class distribution at baseline, month 1, and year 1; (**B**) alterations in NYHA symptom class at first month and first year following transcatheter aortic valve replacement with Portico THV and FlexNav DS.

**Figure 4:**
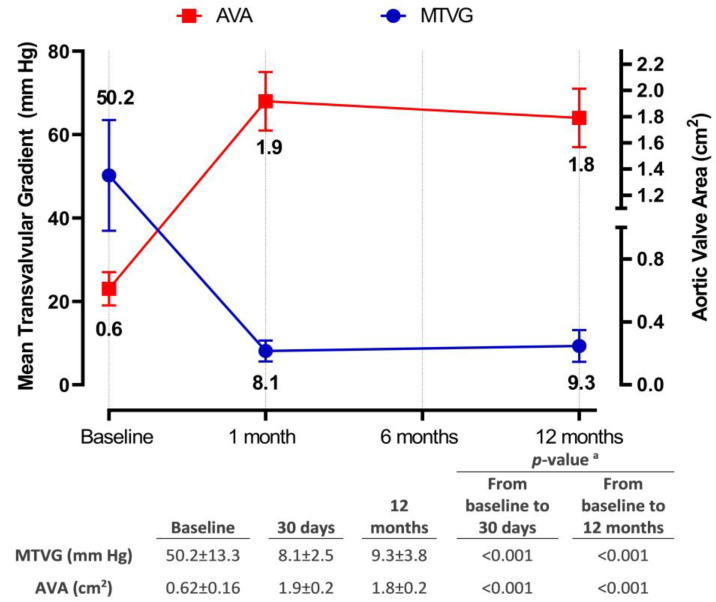
Echocardiographic examination of mean transvalvular gradient and aortic valve area in the postprocedural period. Data are shown as means ± standard deviation. Abbreviations—AVA: aortic valve area, MTVG; mean transvalvular gradient. ^a^ *p*-value of <0.05 was considered statistically significant.

**Figure 5:**
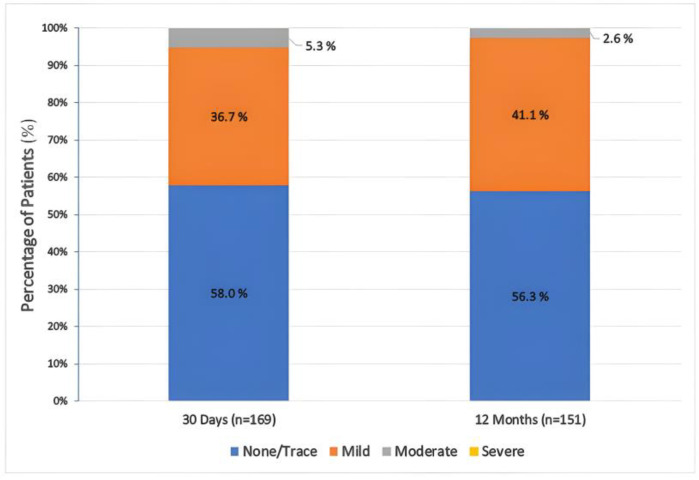
Paravalvular leak rates by severity at the 30th day and 1st year after TAVR.

During post-TAVR follow-up, there was a significant improvement in the NYHA functional class of patients who survived at day 30 (*n* = 161) and year 1 (*n* = 151) compared to the baseline (*n* = 169) cohort. The percentage of patients classified as NYHA class III decreased from 56.2% pre-operatively to 5.0% at day 30 and 5.3% at 1 year, while NYHA class IV decreased from 4.3% pre-operatively to 1.9% at day 30 and 1.3% at 1 year (Figure 3A). Notably, at day 30, 1.2% of patients demonstrated an improvement of at least two functional class levels, while 81.4% showed a one-level improvement. Similarly, at year 1, 0.7% exhibited a two-level or greater improvement, 83.4% showed a one-level improvement, and only 2.0% experienced deterioration (*p* for trend < 0.01, from baseline to 30th day and 12th month) (Figure 3B). Furthermore, there was a significant increase in AVA from 0.62 ± 0.16 cm^2^ at baseline to 1.9 ± 0.2 cm^2^ at day 30 and 1.8 ± 0.2 cm^2^ at year 1. In contrast, MTVG decreased from 50.2 ± 13.3 mm Hg at baseline to 8.1 ± 2.5 mm Hg at day 30 and 9.3 ± 3.8 mm Hg at year 1 (*p* for trend < 0.001, for both; Figure 4). No or mild paravalvular leak (PVL) was observed in 94.7% of patients at day 30 and 97.4% at year 1, whereas the rate of moderate PVLs was 5.3% at day 30 and 2.6% at year 1. Notably, no severe PVL was detected among any of the patients (Figure 5).

## 4. Discussion

The present study documented the procedural, 30-day, and 1-year real-life data for TAVR performed at a single center using the Portico THV and FlexNav DS. Furthermore, Portico THV hemodynamic performance was examined using current VARC-3 criteria in addition to detailed safety and efficacy outcomes. The following are some of the study’s key findings: (i) high procedural success rate, (ii) acceptable hemodynamic results and PVL rates, (iii) low percentage of PPM implantation at 1-year follow-up, and (iv) low 1-year mortality rates. To the best of our knowledge, this is the first real-world study to report short-term outcomes based on the VARC-3 consensus report for patients who underwent TAVR procedures with the Portico THV and FlexNav DS.

### 4.1. Procedural and 30-Day Outcomes

The clinical outcomes with the Portico THV and the next-generation FlexNav DS demonstrated a high rate of procedural success and low occurrence of complications, comparable to other next-generation TAVR systems. Notably, in the present study, the administration of local anesthesia/conscious sedation (LA/CS) was observed in 97% of the TAVR procedures. This utilization rate was higher in earlier investigations involving the Portico THV. For instance, the LA/CS rate was reported as 75.5% in the PORTICO-I trial [20] and 56.7% in a study by Fontana et al. [21]. In the CONFIDENCE Registry, the LA/CS utilization rate was documented as 69.9% in the first-generation delivery system (FG-DS) arm and 82.6% in the FlexNav DS arm [22]. The higher rate observed in the present study can be attributed to several factors, including: (a) a preference for a minimalist TAVR approach whenever feasible, (b) the low profile and high procedural success rate of the FlexNav DS, and (c) the relatively stable hemodynamic conditions achieved during the implantation procedure.

In our study, no fatal intraprocedural complications, such as annular rupture, left ventricular perforation, tamponade, or the need for open-heart surgery, were observed. Specifically, no FlexNav DS-specific issues were identified. It should be noted, however, that annular rupture has been reported in TAVR procedures utilizing self-expandable valves [22,23]. Furthermore, the use of the FlexNav DS with a hydrophilic-coated integrated sheath has been associated with a 49% reduction in major peripheral complications within 30 days compared to the FG-DS [21]. In the present study, the challenges primarily arose from peripheral vascular access, resulting in higher rates of major vascular complications when compared to studies involving the FG-DS and FlexNav DS with the Portico THV (Table S1) [20,21,22,24,25]. Nevertheless, it is important to acknowledge that not all peripheral complications should be directly attributed to the DS technology, as various variables, including female gender, a history of peripheral arterial disease, iliofemoral diameters and calcification, the utilization of vascular closure devices, and the experience of the center, may all contribute to the observed outcomes [26,27,28,29]. In this context, our study population exhibited higher proportions of female participants (68%) and individuals with a history of peripheral arterial disease (17.8%).

Our study demonstrated a 30-day mortality rate comparable to that of other self-expandable THVs [20,21,22,30,31,32,33]. Moreover, our study contributes the first set of Portico THV data including periprocedural mortality data based on the VARC-3 criteria to the existing literature. Concomitant pre-TAVR coronary procedures were performed in 6.5% of cases, which is consistent with previous research findings [22]. Notably, our total procedure time was considerably shorter, which can potentially be attributed to the extensive operator experience with the Portico THV and FlexNav DS.

### 4.2. Permanent Pacemaker Rates

Compared to sAVR, PPM implantation is a more frequent issue associated with TAVR. For instance, the 30-day PPM rates with the Portico THV were reported as 18.7% in the PORTICO-I trial [34] and 15.4% in another study conducted by Fontana et al. [21]. Additionally, the CONFIDENCE Registry [22] found no significant difference between the FlexNav DS (18.9%) and the FG-DS (19.2%) arms in terms of 30-day PPM rates. The FORWARD trial [34] reported 30-day PPM rates of 19.3% for Evolut R (Medtronic, Minneapolis, MN, USA) and 20.7% for Evolut PRO in the FORWARD PRO study [31], both of which are self-expandable THVs. In the case of the ACURATE Neo THV (Boston Scientific, Marlborough, MA, USA), the SAVI-TF registry [35] reported a 30-day PPM rate of 8.3%. As for balloon-expandable THVs, the Myval (Meril Life Science, Vapi, India) exhibited a PPM rate of 7.4%, whereas the Sapien 3 (Edwards Lifesciences, Irvine, CA, USA) demonstrated a PPM rate of 13.4% [36]. In our study, both the 30-day (11.0%) and 12-month PPM rates (12.2%) were relatively low, which is potentially attributable to the operators’ experience, the low radial force, the precise and flexible implantation capabilities of the FlexNav DS, and, consequently, the high level of valve implantation.

### 4.3. Hemodynamic Performance

We observed that the incidence of moderate PVL was 5.3% at the 30-day follow-up and it decreased to 2.6% at 12 months. No patients, however, experienced severe PVL. In the CONFIDENCE Registry [22], the rate of moderate PVL at 30 days was reported as 2.1%, and no cases of severe PVL were observed. The PORTICO-I study with FG-DS [20] reported a rate of 3.9% for moderate-to-severe PVL at 30 days and 2.6% at 1 year. Another study using the FlexNav DS [21] found that the occurrence of moderate-to-severe PVL was 2.6% at discharge and 3.9% at 30 days. Similarly, the FORWARD study with the Evolut R THV [34] reported rates of 2.0% for moderate PVL and 0.1% for severe PVL, and the FORWARD PRO study with the Evolut PRO THV [31] reported a rate of 1.8% for moderate-to-severe PVL. The SAVI TF trial with the ACURATE Neo THV [35] demonstrated a rate of 3.6% for moderate-to-severe PVL at 1 year. Our findings align with these studies in terms of comparable rates of moderate and severe PVL. Some Portico THV results at 30 days and 1 year, with single-digit MTVGs and greater AVAs, indicated positive outcomes. Furthermore, the Portico THV, with its intra-annular design, demonstrated consistent pre- and post-market outcomes similar to widely used supra-annular valves [31,32,33].

### 4.4. One-Year Outcomes

We observed favorable mortality outcomes in TAVR procedures utilizing the Portico THV and the FlexNav DS, with rates comparable to previous studies involving the Portico THV with the FG-DS [20,25], other studies using the FlexNav DS [37], and those involving self-expandable and balloon-expandable valves [32,35]. For instance, Makkar et al. [37] conducted a patient-level pooled analysis of two concurrent prospective, multicenter, pre-market studies (PORTICO IDE (NCT02000115) and the FlexNav EU CE Mark Study (NCT03724812)) and reported a 1-year all-cause mortality rate of 4.7%, which was lower than our findings. However, it is worth noting the limited availability of prospective and real-world data assessing the long-term performance of the Portico THV and FlexNav DS (Table S1).

We found the 30-day and 1-year neurologic event rates to be 3.0% and 7.7%, respectively. For 30-day results, the CONFIDENCE Registry [22] indicated a frequency of neurological events of 3.2% in the FlexNav DS arm and 3.6% in the FG-DS arm. In a FlexNav DS study by Fontana et al. [21], the rate was reported as 3.9%. Briefly, prior research has found that neurological events occur at a rate of 2 to 5% after one year of follow-up [20]. Among various self-expandable valve trials, the rate of stroke/TIA on day 30 in the Surgical Replacement and Transcatheter Aortic Valve Implantation (SURTAVI) study [6] with the Evolut-R THV was 4.5%, and it was 2.3% in the SAVI-TF Registry [35], which reported 1-year results for ACURATE Neo THV implantation. Variations in these rates could be attributed to limitations and/or uncertainty (disabling and non-disabling stroke) in classifying stroke/TIA with the earlier standard VARC-2 criteria [9]. The VARC-3 consensus, on the other hand, recategorized acute neurological episodes (NeuroARC types 1a, 1b, 1c, 1d, and 1e; 2a and 2b; and 3a and 3b) with grades (mild, moderate, and severe) using the National Institutes of Health Stroke Scale (NIHSS) [38] and timings (acute, sub-acute, early, and late) [12]. Even though neurological events could not be categorized according to severity, all but NeuroARC type 3b were included in the present analysis. It is also important to keep in mind that numerous other factors, such as a history of cerebrovascular events [39], the presence of AF [40,41], the use of oral anticoagulants and antiplatelets [42], impaired kidney function [39,40], peripheral artery disease [39], carotid artery disease [43], and intracardiac thrombus [44], may influence the frequency of neurological events in TAVR. For these reasons, the rate of neurological events may have been higher in our study than in other studies reported using VARC-2. Similarly, the present study’s higher rate of bleeding compared to prior studies could in part be attributed to the higher usage of anticoagulation, heterogeneity induced by insufficient classification in the previous consensus, and the high stroke frequency.

### 4.5. Study Limitations

The present study contains some shortcomings. Most importantly, it was designed in a retrospective and single-center manner. It should be emphasized, nonetheless, that the study data were gathered prospectively due to the study center’s registration criteria. Also, compared to previous multicenter trials, the study population can be deemed sufficient for a single center. However, due to the low number of events such as fatality, cardiovascular, and neurological events, a larger patient sample may have produced stronger statistical results. The absence of an FG-DS arm was another major drawback.

## 5. Conclusions

The present study presented real-world data regarding the safety and efficacy outcomes of TAVR utilizing the Portico THV and the next-generation, low-profile FlexNav DS. The findings demonstrated favorable safety and efficacy outcomes. However, it is important to acknowledge that the utilization of distinct endpoints and classifications, such as the VARC-3 criteria, in this study compared to the existing literature could have potentially influenced the reported rates of acute kidney injury and major vascular complications. Consequently, future trials adopting the current endpoints are essential to attain more precise and comparable results.

## Figures and Tables

**Table 1:** Demographic and baseline characteristics (*n* = 169).

Characteristic	
Age, years	75.8 ± 7.7
Gender, female, *n* (%)	115 (68.0)
Coronary artery disease, *n* (%)	68 (40.2)
Previous CABG, *n* (%)	45 (26.6)
Previous coronary stenting, *n* (%)	39 (23.1)
Prior atrial fibrillation, *n* (%)	30 (17.8)
Previous mitral valve prosthesis, *n* (%)	3 (1.8)
NYHA class, *n* (%)	
I	18 (10.7)
II	48 (28.4)
III	95 (56.2)
IV	8 (4.7)
Diabetes mellitus, *n* (%)	48 (28.4)
Hypertension, *n* (%)	91 (53.8)
Prior neurological events, *n* (%)	11 (6.5)
Peripheral artery disease, *n* (%)	30 (17.8)
Permanent pacemaker, *n* (%)	6 (3.6)
Smoking, *n* (%)	33 (19.5)
Chronic lung disease, *n* (%)	23 (13.6)
Anticoagulant use, *n* (%)	19 (11.2)
Chronic kidney disease, *n* (%)	25 (14.8)
Chronic liver disease or cirrhosis, *n* (%)	3 (1.8)
History of malignancy, *n* (%)	5 (3.0)
STS PROM score ^1^, %	4.63 ± 1.73
Low risk (<4.0%)	53 (31.4)
Moderate risk (4.0–7.9%)	101 (59.8)
High risk (≥8.0%)	15 (8.9)
Laboratory Parameters	
Creatinine, mg/dL	0.96 ± 0.48
e-GFR ^2^, mL/min/1.73 m^2^	68 ± 22
LDL-C, mg/dL	118 ± 51
HDL-C, mg/dL	48 ± 15
Total cholesterol, mg/dL	187 ± 31
Glucose, mg/dL	124 ± 42
NT-proBNP, pg/mL	1110 (374–3925)
WBC, 10^3^/µL	7.5 ± 3.1
Hemoglobin, mg/dL	11.5 ± 1.8
Platelet count, 10^3^/µL	224 ± 73
hs-cTnI, pg/mL	16 (3–32)
Echocardiographic Parameters	
Left ventricular ejection fraction, %	53.9 ± 10.2
Left ventricular systolic dysfunction ^3^, *n* (%)	38 (22.5)
Mean transvalvular aortic gradient, mm Hg	50.2 ± 13.3
Maximum transvalvular aortic gradient, mm Hg	74.8 ± 19.2
Maximum aortic valve velocity, m/s	4.33 ± 0.57
Aortic valve area, cm^2^	0.62 ± 0.16
Aortic valve area/BSA, cm^2^/m^2^	0.34 ± 0.10
Bicuspid aortic valve, *n* (%)	29 (17.2)
Mitral insufficiency (moderate-to-severe), *n* (%)	22 (13.0)
Mitral stenosis (moderate-to-severe), *n* (%)	15 (8.8)
Aortic insufficiency (moderate-to-severe), *n* (%)	54 (32.0)
Tricuspid insufficiency (moderate-to-severe), *n* (%)	32 (18.9)
e-PASP on tricuspid regurgitation, mm Hg	33 ± 11

^1^ Calculated via https://www.sts.org/resources/risk-calculator (accessed on December 2021); ^2^ calculated according to the Chronic Kidney Disease Epidemiology Collaboration (CKD-EPI) equation; ^3^ left ventricular systolic dysfunction LV EF < %50. Data are shown as *n* (%), median (interquartile range; 25th–75th percentiles), and mean ± standard deviation. Abbreviations—BSA: body surface area, BNP: brain natriuretic peptide, CABG: coronary artery bypass surgery, e-GFR: estimated glomerular filtration rate, e-PASP: estimated pulmonary artery systolic pressure, HDL-C: high-density lipoprotein cholesterol, hs-cTnI: high-sensitivity cardiac troponin I, LDL-C: low-density lipoprotein cholesterol, NYHA: New York Heart Association, STS PROM: Society of Thoracic Surgeons Predicted Risk of Mortality, WBC: white blood cell.

**Table 2:** Periprocedural features.

Characteristic	
Anesthesia, *n* (%)	
General	5 (3.0)
Conscious sedation/local anesthesia	164 (97.0)
Portico THV size, *n* (%)	
23 mm	6 (3.5)
25 mm	24 (14.2)
27 mm	38 (22.5)
29 mm	101 (59.8)
Pre-dilatation, *n* (%)	135 (79.8)
Post-dilatation, *n* (%)	89 (52.6)
Total procedure time ^1^ (min)	58.8 ± 12.8
Fluoroscopy time (min)	25.8 ± 6.1
Contrast volume (mL)	232 ± 58
Need for extra-stiff wire ^2^, *n* (%)	2 (1.2)
Access route, *n* (%)	
Right transfemoral	142 (94.1)
Left transfemoral	7 (4.1)
Left subclavian/axillary	3 (1.8)
Any resheathing performed, *n* (%)	142 (84.0)
One resheath	55/142 (38.7)
Two resheaths	48/142 (33.8)
Three or more resheaths	39/142 (27.5)
Intraprocedural mortality, *n* (%)	0 (0.0)
Conversion to open-heart surgery, *n* (%)	0 (0.0)
Annular rupture, *n* (%)	0 (0.0)
Correct positioning of single Portico THV, *n* (%)	162 (95.9)
Need for second Portico THV, *n* (%)	7 (4.1)
25 mm	1/7 (14.3)
29 mm	6/7 (85.7)
Coronary obstruction ^3^, *n* (%)	1 (0.6)
>2 units ES replacement ^4^, *n* (%)	20 (11.8)
Length of hospital stay, days	4 (2–5)
Technical outcomes, *n* (%)	
Concomitant coronary intervention before TAVR	11 (6.5)
Successful access, delivery, and implantation of the Portico THV with FlexNav DS	169 (100.0)
Procedural success ^5^	162 (95.9)
Technical success ^6^	150 (88.8)

^1^ First incision to closure time; ^2^ use of a stiffer guidewire for conditions such as peripheral tortuosity and a sharp arcus aortic angle (e.g., Back-Up Meier); ^3^ transient coronary obstruction due to balloon pre-dilatation; ^4^ post-operative replacement during the index procedure hospitalization; ^5^ includes successful peripheral vascular access, single THV placement in the exact location, and DS retrieval; ^6^ in addition to procedural success, defined as the lack of procedural mortality and peripheral/structural/device-related challenges necessitating surgical/interventional intervention (excluding permanent pacemaker implantation), as per VARC-3 criteria. Data are shown as *n* (%), n/N (%), or median (interquartile range; 25th–75th percentiles). Abbreviations—DS: delivery system, ES: erythrocyte suspension, TAVR: transcatheter aortic valve replacement, THV: transcatheter heart valve.

**Table 3:** Thirty-day outcomes according to VARC-3 criteria (*n* = 169).

Outcomes	
All-cause mortality	8 (4.7)
Cardiovascular mortality	6 (3.6)
Neurological events ^1^	5 (3.0)
Myocardial infarction	3 (1.8)
Periprocedural (≤48 h after TAVR procedure) ^2^	2 (1.2)
Spontaneous (>48 h after TAVR procedure) ^3^	1 (0.6)
Acute kidney injury	25 (14.8)
Stage 1	20 (11.8)
Stage 2 or 3	3 (1.8)
Stage 4	2 (1.2)
Bleeding ^4^	21 (12.4)
Type 2	13 (7.7)
Type 3	8 (4.7)
Type 4	0 (0.0)
Overall pacemaker implantation ^5^	18 (10.7)
New pacemaker implantation ^6^	18 (11.0)
New-onset atrial fibrillation	12 (7.1)
New-onset LBBB	5 (3.0)
Pericardial tamponade	0 (0.0)
Vascular and access site-related outcomes, *n* (%)	
No peripheral or access site-related complications	147 (87.0)
Overall vascular complications	22 (13.0)
Major vascular complications	19 (11.2)
Major non-FlexNav DS site complications	6 (3.6)
Iliofemoral dissection	4 (2.4)
Inguinal hematoma (moderate-to-severe)	12 (7.1)
Pseudoaneurysm (moderate-to-severe)	6 (3.6)
Major percutaneous closure device failure	2 (1.2)
Percutaneous vascular-covered stent implantation	5 (3.0)
İliofemoral arteries	4/5 (80.0)
Left subclavian/axillary	1/5 (20.0)
Requiring unplanned peripheric surgery	1 (0.6)

^1^ According to the VARC-3 criteria, NeuroARC type-1, type-2, and type-3a events were included, while NeuroARC type-3b was excluded; ^2^ criteria for TAVR-related MI ≤48 h after the index procedure, type-5 MI; ^3^ according to VARC-3 criteria, type-1 MI; ^4^ Bleeding Academic Research Consortium (BARC) classification: type-1 (minor), type-2 (major), type-3 (life-threatening), and type-4 (leading to death) bleeding. Type-1 (minor) bleeding was excluded; ^5^ among patients regardless of pacemaker at baseline; ^6^ among patients without pacemaker at baseline. Data are shown as *n* (%) and n/N (%). Abbreviations—DS: delivery system, ES: erythrocyte suspension, LBBB: left bundle branch block, TAVR: transcatheter aortic valve replacement, THV: transcatheter heart valve.

**Table 4:** Endpoints from 31 days to 12 months and at 12 months according to VARC-3 criteria (*n* = 169).

	31 Days to 12 Months	Total (12 Months)
Primary Endpoints, *n* (%)		
All-cause mortality	10 (5.9)	18 (10.7)
Cardiovascular mortality	8 (4.7)	14 (8.3)
Neurological events ^1^	8 (4.7)	13 (7.7)
Secondary Endpoints, *n* (%)		
Myocardial infarction	0 (0.0)	3 (1.8)
Acute kidney injury ^2^	4 (2.4)	29 (17.2)
Bleeding ^3^	5 (3.0)	26 (15.4)
Overall pacemaker implantation ^4^	2 (1.1)	20 (11.8)
New pacemaker implantation ^5^	2 (1.2)	20 (12.2)
New-onset atrial fibrillation	3 (1.8)	15 (8.9)
Overall vascular complications	0 (0.0)	22 (13.0)
Major vascular complications ^6^	0 (0.0)	13 (7.7)
Pericardial tamponade	0 (0.0)	0 (0.0)

^1^ According to the VARC-3 criteria, NeuroARC type-1, type-2, and type-3a events were included, while NeuroARC type-3b was excluded; ^2^ all events (types 1–4) were included; ^3^ Bleeding Academic Research Consortium (BARC) classification: type-1 (minor), type-2 (major), type-3 (life-threatening), and type-4 (leading to death) bleeding. Type-1 (minor) bleeding was excluded; ^4^ among patients regardless of pacemaker at baseline; ^5^ among patients without pacemaker at baseline; ^6^ delivery system site-associated major vascular event rate. Data are shown as *n* (%).

**Table 5:** Causes of cardiovascular death at 30 days and 12 months and timing of all-cause mortality (*n* = 169).

	30 Days	12 Months
Main Causes of Cardiovascular Death, *n* (%)		
Intraprocedural mortality	0 (0.0)	0 (0.0)
Heart failure	1 (0.6)	3 (1.8)
Myocardial infarction	0 (0.0)	0 (0.0)
Stroke	1 (0.6)	2 (1.2)
Vascular access site-related complication	1 (0.6)	1 (0.6)
Bleeding	2 (1.2)	3 (1.8)
Cardiac tamponade	0 (0.0)	0 (0.0)
Sudden cardiac death	1 (0.6)	3 (1.8)
Death of unknown cause	0 (0.0)	2 (1.2)
Valve-related mortality, *n* (%)	0 (0.0)	0 (0.0)
Timing of All-Cause Mortality, *n* (%)		
Periprocedural mortality	9 (5.3)	
≤30 days after the TAVR procedure	8 (4.7)	
>30 days but during hospitalization after TAVR ^1^	1 (0.6)	
Early mortality ^2^	10 (5.9)	

^1^ Mortality at index hospitalization without discharge after TAVR; ^2^ death occurring >30 days but ≤1 year after the TAVR. Data are shown as *n* (%). Abbreviations—TAVR: transcatheter aortic valve replacement.

## Data Availability

Data are available on request due to privacy and ethical restrictions.

## Supplementary Materials

The following supporting information can be downloaded at: https://www.mdpi.com/article/10.3390/jcm12165373/s1. Table S1: Comparison of designs and major results of known Portico THV studies [20,21,22,24,25,37,45].
