# Supplementary material for: Real-Life Performance and Clinical Outcomes of Portico Transcatheter Aortic Valve with FlexNav Delivery System: One-Year Data from a Single-Center Experience
Source: J Clin Med. 2023 Aug 18;12(16):5373. doi: 10.3390/jcm12165373 (PMC10455755; doi:10.3390/jcm12165373)
Supplement: Supplementary file 1 [file jcm-12-05373-s001.zip › jcm-2475089-supplementary.pdf]

Supplementary Materials

Table S1. Comparison of design and major results of known Portico valve studies.

	**Denegri et al. [45] (2018)**	**CONFIDENCE Registry [22] (2022)**	**Fontana et al. [21] (2020)**	**PORTICO-I Trial [20] (2018)**	**PORTICO-I Trial [20] (2018)**	**Linke et al. [25] (2018)**	**PORTİCO IDE [24] (2020)**	**Makkar et al. [37] (2022)**	**Our Study (2023)**
**Patient Group**	73 (first-generation DS)	500 (FlexNav)/501 (first-generation DS)	180 (All FlexNav DS)	941 (first-generation DS)	941 (first-generation DS)	222 (first-generation DS)		193 (All FlexNav DS)	169 (All FlexNav DS)
**Design**	Single center, Real World	Multi-center, Real World	Multi-center, Pre-market (PORTICO IDE n=137, FlexNav EU CE Mark Study n=46)	Multi-center	Multi-center	Multi-center, Pre-CE Mark study	Multi-center FDA-approved trial	Multi-center, Pre-market (PORTICO IDE n=147, FlexNav EU CE Mark Study n=47)	Single-center, Real World
**Follow-up**	30 days	30 days	30 days	30 days	1 year	1 year	1 year	1 year	1 year
**End-points**	VARC-2	VARC-2	VARC-2	VARC-2	VARC-2	VARC-2	VARC-2	VARC-2	VARC-3
**All-cause mortality, n (%)**	2.7	2.0	0.6	2.7	12.1	13.8	14.3	4.7	11.2
**Cardiovascular mortality, n (%)**	--------	1.2	0.6	2.4	6.6	9.4	---	---	8.3
**All stroke/TIA, n (%)**	2.7	3.2	3.9 (all stroke/TIA)	2.6 (TIA except)	5.2 (all stroke/TIA)	5.8 (disabling stroke)	1.6 (disabling stroke)	2.1(disabling stroke)	7.7(all neurological event)
**New PPM, n (%)**	12.0	18.9	15.4	18.7	19.5	14.7	---	18.4	12.2
**Need for second valve, n (%)**	2.8	2.0	3.3	2.0	2.0	---	---	---	4.1
**Major vascular complications, n (%)**	4.7	8.2	5.0	5.5	5.7	8.8	9.6	----	7.7
**Acute kidney injury, n (%)**	2.7	0.8	0.0 (stage 2-3)	3.4	4.2 (stage 2-3)	3.0 (stage 3)	---	----	17.2 (All stage)
**MTVG, mm Hg**	6.9±3.2	7.1	7.1±3.2	8.6±3.9	8.4±0.61	8.4±0.61	---	7.4±4.3	9.3±3.8
**AVA, cm²**	---	1.82	1.77±0.41	1.79±0.50	1.74±0.03	1.74±0.03	---	1,76±0,43	1.8±0.2

**Moderate-to-severe PVL, n (%)**	---	2.1	3.9	2.1	7.5	5	7.8	---	2.6
**Total procedure time, min**	---	73.4±39.8	49.1±21.9	76.5±35.0	---	----	---	---	58.8±12.8

Data are shown in n (%), and mean± standard deviation. Abbreviations; AVA: aortic valve area, DS: delivery system, MTVG: mean transvalvular gradient, PPM: permanent pacemaker, PVL: paravalvular leak, TIA: transient ischemic attack, VARC: the Valve Academic Research Consortium.
